# A randomized double-blind clinical trial on safety and efficacy of tauroursodeoxycholic acid (TUDCA) as add-on treatment in patients affected by amyotrophic lateral sclerosis (ALS): the statistical analysis plan of TUDCA-ALS trial

**DOI:** 10.1186/s13063-023-07638-w

**Published:** 2023-12-05

**Authors:** Flavia L. Lombardo, Stefania Spila Alegiani, Flavia Mayer, Marta Cipriani, Maria Lo Giudice, Albert Christian Ludolph, Christopher J. McDermott, Philippe Corcia, Philip Van Damme, Leonard H. Van den Berg, Orla Hardiman, Gabriele Nicolini, Nicola Vanacore, Brian Dickie, Alberto Albanese, Maria Puopolo, TUDCA-ALS Study Group, Paolo Tornese, Antoniangela Cocco, Michela Matteoli, Eliana Lauranzano, Maria Luisa Malosio, Chiara Adriana Elia, Adriano Chiò, Umberto Manera, Cristina Moglia, Andrea Calvo, Paolina Salamone, Giuseppe Fuda, Carlo Colosimo, Cristina Spera, Prabha Cristina Ranchicchio, Giuseppe Stipa, Domenico Frondizi, Christian Lunetta, Valeria Sansone, Claudia Tarlarini, Francesca Gerardi, Vincenzo Silani, Alberto Doretti, Eleonora Colombo, Gianluca Demirtzidis, Gioacchino Tedeschi, Francesca Trojsi, Carla Passaniti, Stefania Ballestrero, Johannes Dorst, Ulrike Weiland, Andrea Fromm, Maximilian Wiesenfarth, Katharina Kandler, Simon Witzel, Markus Otto, Joachim Schuster, Thomas Meyer, André Maier, Dagmar Kettemann, Susanne Petri, Lars Müschen, Camilla Wohnrade, Anastasia Sarikidi, Alma Osmanovic, Julian Grosskreutz, Annekathrin Rödiger, Robert Steinbach, Benjamin Ilse, Uta Smesny, Robert Untucht, René Günther, Maximilian Vidovic, Pamela Shaw, Alexis Collins, Helen Wollff, Theresa Walsh, Lee Tuddenham, Mbombe Kazoka, David White, Stacy Young, Benjamin Thompson, Daniel Madarshahian, Suresh K. Chhetri, Amina Chaouch, Carolyn A. Young, Heike Arndt, Coliver Hanemann, Thomas Lambert, Stephane Beltran, Philippe Couratier, Florence Esselin, William Camu, Elisa De La Cruz, Gwendal Lemasson, Pegah Masrori, Sinead Maguire, Liz Fogarty, Toyosi Atoyebi, Niamh Ní Obáin

**Affiliations:** 1 National Centre for Disease Prevention and Health Promotion, Italian National Institute of Health, Rome, Italy; 2 National Center for Drug Research and Evaluation, Italian National Institute of Health, Rome, Italy; 3 Department of Statistical Sciences, Sapienza University of Rome, Rome, Italy; 4 Department of Neuroscience, Italian National Institute of Health, Rome, Italy; 5 Neurology Department, IRCCS Humanitas Research Hospital, Rozzano, Italy; 6 Neurology Department, University of Ulm, Ulm, Germany; 7 German Centre of Neurodegenerative Diseases, Site Ulm, Ulm, Germany; 8 Department of Neuroscience, Sheffield Institute for Translational Neuroscience, University of Sheffield, Sheffield, UK; 9 Centre de Référence Maladie Rare (CRMR) SLA Et Les Autres Maladies du Neurone Moteur (FILSLAN), Tours, France; 10 CHU Bretonneau, Tours, France; 11 Federation des CRMR-SLA Tours-Limoges, LITORALS, Tours, France; 12 Faculté de Médecine, INSERM U1253, “iBrain,” Université François-Rabelais de Tours, Tours, France; 13 Neurology Department, University Hospitals Leuven, Louvain, Belgium; 14 Neuroscience Department, KU Leuven, Louvain, Belgium; 15 Department of Neurology, UMC Utrecht Brain Center, University Medical Centre Utrecht, Utrecht, Netherlands; 16 Academic Unit of Neurology, Trinity Biomedical Sciences Institute, Dublin, Ireland; 17 Clinical Research Centre, Beaumont Hospital, Dublin, Ireland; 18 Medical Affairs, Bruschettini S.R.L, Genoa, Italy; 19 Motor Neurone Disease Association, Northampton, UK

**Keywords:** Statistical analysis plan, Randomized, Double-blind, Clinical trial, Phase III, Amyotrophic lateral sclerosis, Bile acids

## Abstract

**Background:**

Amyotrophic lateral sclerosis (ALS) is a highly debilitating neurodegenerative condition. Despite recent advancements in understanding the molecular mechanisms underlying ALS, there have been no significant improvements in therapeutic options for ALS patients in recent years. Currently, there is no cure for ALS, and the only approved treatment in Europe is riluzole, which has been shown to slow the disease progression and prolong survival by approximately 3 months. Recently, tauroursodeoxycholic acid (TUDCA) has emerged as a promising and effective treatment for neurodegenerative diseases due to its neuroprotective activities.

**Methods:**

The ongoing TUDCA-ALS study is a double-blinded, parallel arms, placebo-controlled, randomized multicenter phase III trial with the aim to assess the efficacy and safety of TUDCA as add-on therapy to riluzole in patients with ALS. The primary outcome measure is the treatment response defined as a minimum of 20% improvement in the ALS Functional Rating Scale-Revised (ALSFRS-R) slope during the randomized treatment period (18 months) compared to the lead-in period (3 months). Randomization will be stratified by country. Primary analysis will be conducted based on the intention-to-treat principle through an unadjusted logistic regression model. Patient recruitment commenced on February 22, 2019, and was closed on December 23, 2021. The database will be locked in September 2023.

**Discussion:**

This paper provides a comprehensive description of the statistical analysis plan in order to ensure the reproducibility of the analysis and avoid selective reporting of outcomes and data-driven analysis. Sensitivity analyses have been included in the protocol to assess the impact of intercurrent events related to the coronavirus disease 2019. By focusing on clinically meaningful and robust outcomes, this trial aims to determine whether TUDCA can be effective in slowing the disease progression in patients with ALS.

**Trial registration:**

ClinicalTrials.gov NCT03800524. Registered on January 11, 2019.

**Supplementary Information:**

The online version contains supplementary material available at 10.1186/s13063-023-07638-w.

## Introduction

### Background and rationale

Amyotrophic lateral sclerosis (ALS) is a chronic non-communicable neurodegenerative rare disease that affects approximately 40,000 individuals in Europe, leading to around 11,000 deaths each year [1, 2]. Despite much has been achieved over the last two decades in understanding the disease complexity, there is an urgent need to find disease-modifying therapies that can slow disease progression and extend patient survival. Currently, the only approved treatment in Europe is riluzole, a glutamate release inhibitor, which provides a modest extension of survival in ALS patients. Riluzole has been shown to increase survival by only approximately 3 months [3], but this indicates that modifying disease progression is a realistic goal. Nonetheless, all subsequent drug trials for ALS have failed to deliver improvements in patient care [4].

The ongoing “Safety and efficacy of tauroursodeoxycholic acid as add-on treatment in patients affected by amyotrophic lateral sclerosis” (TUDCA-ALS) study takes advantage of the results of a recent proof-of-concept phase IIb study [5] showing that, in patients who received tauroursodeoxycholic acid (TUDCA) in addition to riluzole, the per-year decline rate of ALS functional rating scale revised (ALSFRS-R) was of about 7 points smaller (on a 0–48 score) compared to riluzole only. The efficacy of TUDCA is further supported by the evidence that TUDCA has cytoprotective properties in animal models of neurodegenerative diseases [6]. Additionally, several clinical studies have shown the safety and tolerability of TUDCA. Therefore, this large-scale, phase III, TUDCA-ALS trial aims to confirm and further evaluate the efficacy of TUDCA as a disease-modifying agent in ALS. The study also incorporates the assessment of reliable biomarkers associated with disease progression and cytoprotective activity during the 18-month treatment period.

### Study objectives

The primary objective of the TUDCA-ALS study is to assess the efficacy of TUDCA, as an add-on therapy to riluzole, in slowing down the progression of ALS throughout the 18-month treatment period, compared to the lead-in phase. The secondary objectives of this study are as follows: (1) to assess the efficacy of TUDCA in slowing disease progression and functional impairment in patients with ALS, as measured by the survival time, the ALSFR-R, the ALS Assessment Questionnaire-40 (ALSAQ-40) and EuroQol 5-Dimension 5L questionnaire (EQ-5D-5L), forced vital capacity (FVC), and the Medical Research Council (MRC) sum-score for muscle force, and (2) to evaluate the long-term safety and tolerability of TUDCA.

The exploratory objective of this study is to investigate the effect of TUDCA on biomarkers associated with disease progression, such as neurofilaments in cerebrospinal (CSF) fluid and serum, serum expression of matrix metalloproteinase-9 (MMP-9) in serum, and plasma creatinine levels.

This document describes in detail the predetermined statistical analysis plan (SAP) for the TUDCA-ALS trial.

## Methods

The analysis outlined in this document is in full compliance with International Conference on Harmonisation ICH E9 guidance [7] and follows the guidelines for the content of statistical analysis plans in clinical trials [8] (Additional file 1).

The SAP (version 2.0 of April 05, 2023) is an update of SAP version 1.3 dated 23 July 2019 and includes the sample size reassessment and additional analyses to account for the COVID-19 pandemic, in alignment with the most recent protocol (version 2.0, 23 July 2021). The SAP has been finalized prior to the completion of the data collection. The study protocol has been registered at www.clinicaltrials.gov (NCT03800524). The updated version of the protocol, providing detailed information on background, design, and rationale, has been published elsewhere [9]. The trial is being conducted in accordance with the Helsinki declaration and the local laws and regulations of the respective countries, prioritizing the highest level of patient protection. The protocol was approved by all the involved ethics committees and by the national regulatory authorities.

### Trial design

This is a phase III, multicenter, randomized, double-blind, placebo-controlled, parallel-group study. Eligible patients are randomly assigned to one of two treatment arms: TUDCA or placebo. The treatment is administered by oral route, as an add-on to the standard therapy with riluzole, at the dose of 50 mg twice daily (100 mg daily).

Patient randomization takes place following a lead-in period of 12 weeks (3 months), during which three assessments are conducted at 6-week intervals. Throughout the subsequent 18-month double-blind phase, clinical assessments are performed every 3 months. This design enables the measurement of disease progression both before and after the initiation of treatment (either active or placebo). The investigational medicinal product is administered orally at the dose of 1 g twice daily (2 g daily) for 18 months in addition to riluzole. Patients are included in the trial only after providing written informed consent. The trial is conducted in 25 clinical centers located in Belgium, France, Italy, Germany, Netherlands, Ireland, and the UK.

### Randomization

Patients are randomized 1:1 to receive TUDCA or placebo. After completing the lead-in period at month 0, eligible patients are randomized by means of a computerized central randomization system and coupled to a unique treatment code through an electronic platform. The randomization list has been generated using STATA software with the “ralloc” command. A permuted block randomization procedure, stratified by country, has been adopted to ensure balance across treatment groups.

### Sample size

The study was powered to assess the potential benefit of TUDCA compared with placebo on the rate of progression, as measured by the ALS-FRS-R total score. The target number of participants was determined based on the expected proportion of responders. In the original planning, based on data from the phase II study [5], a sample size of 199 subjects per arm was calculated to detect a 10% difference between arms assuming a response rate of 10% in the placebo group. This sample size provides at least 80% power with a two-sided alpha of 5%. Considering a dropout rate of 10% at 18 months, a total sample size of 440 patients was planned. Such a sample size provides 83.3% power to detect a 13% absolute difference in survival (defined as death or respiratory insufficiency) between the two treatment groups, corresponding to a hazard ratio of 0.616, assuming an 18-month survival of 60% in the placebo group. However, due to the impact of the COVID-19 outbreak on recruitment [9], the enrolment was planned to be closed at 320 randomized patients. The reassessed sample size allows us to detect an absolute difference of 11% in the proportion of responders between the TUDCA and the placebo arms. It also provides a power of 80% to detect a 14% absolute difference in survival, corresponding to a hazard ratio of 0.58.

### Framework

The aim of the study is to assess the superiority of TUDCA over placebo in terms of efficacy and safety parameters.

### Statistical interim analyses and stopping guidance

An interim analysis was originally scheduled only to verify the hypotheses specified in the study power calculation and to increase the sample size if needed. Nevertheless, due to the reassessment of the sample size resulting from the implementation of COVID-19 mitigation strategies, the interim analysis was consequently canceled.

### Timing of final analysis and outcome assessment

**Fig. 1:**
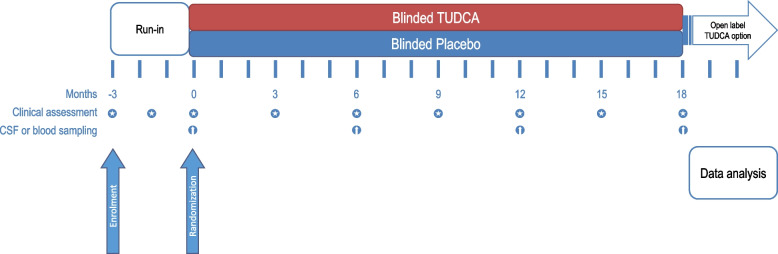
Timeline of the study and visit schedule

The final analysis will be conducted upon completion of the final visit of the last enrolled patient (Fig. 1). Blinding will be removed when all analyses have been performed, following a triple-blind scheme. The schedule for assessing outcomes is presented in Table 1.

**Table 1:** Outcome assessment

Visit	Lead-in			Double-blind trial					
Timescale	M-3	W-6	M0	M3	M6	M9	M12	M15	M18
ALSFRS-R	✓	✓	✓	✓	✓	✓	✓	✓	✓
ALSAQ-40 questionnaire	✓	✓	✓	✓	✓	✓	✓	✓	✓
FVC	✓	✓	✓	✓	✓	✓	✓	✓	✓
EQ-5D-5L questionnaire	✓	✓	✓	✓	✓	✓	✓	✓	✓
MRC sum-score	✓	✓	✓	✓	✓	✓	✓	✓	✓
Survival or respiratory insufficiency	✓	✓	✓	✓	✓	✓	✓	✓	✓
Blood biomarkers			✓		✓		✓		✓
CSF biomarkers			✓				✓		
Safety parameters	✓	✓	✓	✓	✓	✓	✓	✓	✓

*M* month, *W* week

## Statistical principles

Statistical significance will be determined by a two-sided *p*-value ≤ 0.05. Confidence intervals at the 95% level (95% CI) will be reported for all outcomes and measures of association (proportions, means, odds ratios and hazard ratios). Main analyses will be carried out on the intention-to-treat (ITT) population.

### Adherence and protocol deviations

The clinical sites will adopt all reasonable measures to ensure data collection in accordance with the protocol. However, practical circumstances may lead to some variations beyond the site’s control. Such deviations will be documented and summarized, along with the reason for their occurrence. Adherence to the study protocol is based on the proportion of completed visits to those scheduled and the proportion of scheduled doses that were consumed.

### Analysis population

ITT population is defined as all patients who were randomized into the study.

The per-protocol population includes those patients adherent to both visit and medication, respectively defined as patients who completed at least 80% of the expected routine study visits and consumed at least 80% of the prescribed assigned medication [10, 11].

Following the COVID-19 amendment (March 23, 2020), telemedicine visits were introduced as an alternative to in-person visits. For the per-protocol analysis purpose, these two modalities will be considered exchangeable.

The safety population (full analysis population) includes all subjects participating in the trial who received at least one dose of the trial medication. The safety population does not include subjects who dropped out before receiving any treatment.

## Trial population

### Eligibility

Eligible candidates are patients of any gender, aged 18 to 80 years, with probable or probable laboratory-supported, or definite ALS diagnosis according to the revised El Escorial ALS diagnostic criteria [12], having disease duration no longer than 18 months, vital capacity at least 70% of normal, stable on riluzole treatment for 3 months during the lead-in period, and have provided signed informed consent. The main exclusion criteria include cognitive impairment or psychiatric illness, active peptic ulcer, previous surgery, or infections of the small intestine. Detailed inclusion and exclusion criteria can be found in the study protocol [9]. All eligibility criteria must be met by the time of the randomization visit (month 0).

### Recruitment

**Fig. 2:**
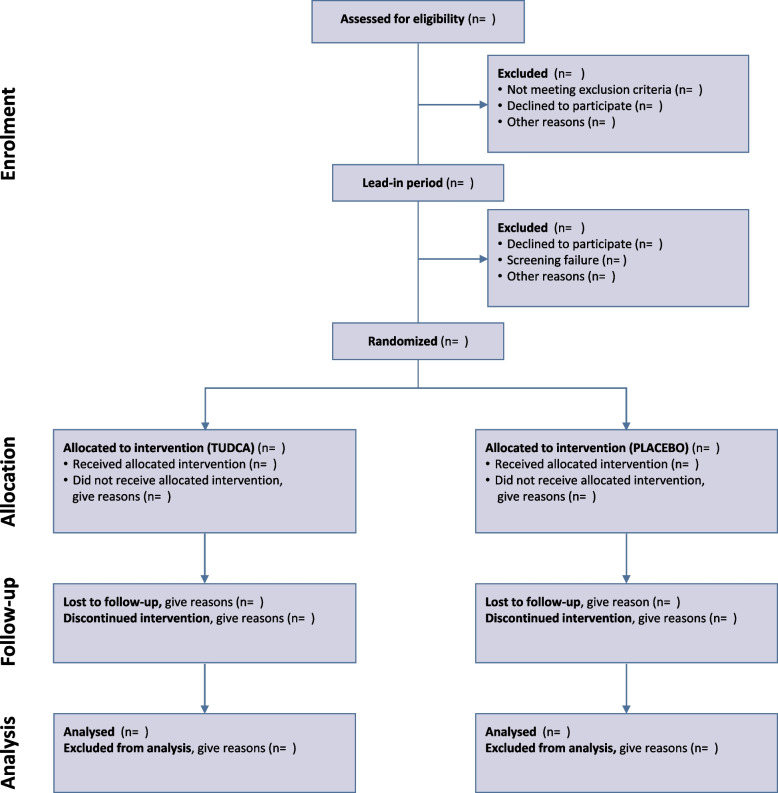
TUDCA-ALS flow diagram (CONSORT 2010)

The flow of study participants, including the number of subjects assessed for eligibility, excluded, randomized, allocated to treatment, withdrawn, or lost to follow-up, will be summarized in a Consolidated Standards of Reporting Trials (CONSORT) diagram (Fig. 2).

### Withdrawal/follow-up

Patients may withdraw from this study at any time. In case of withdrawal, research samples and data will continue to be stored and analyzed for future research, unless the patient specifically requests the withdrawal of samples. The timing and reasons of withdrawal and/or lost to follow-up (LTF) will be summarized, whenever possible, as time to event data and will be presented using Kaplan–Meier curves, both overall and by treatment group.

### Baseline patient characteristics

Patients will be described with respect to all variables collected at baseline (month 0, Fig. 1), separately for the two randomized groups. These variables include demographic information, medical and disease history, physical examination, vital signs, electrocardiogram results, FVC, ALSFRS-R, MRC sum-score, EQ-5D-5L, ALSAQ-40, hematological and biochemical parameters, and concomitant treatments. Categorical data will be summarized by numbers and percentages. Continuous data will be summarized by mean, standard deviation, range, median, and interquartile range. Tests for statistical significance will not be conducted for baseline characteristics; instead, any imbalances will be noted based on their clinical importance.

## Analysis

### Outcomes

The primary outcome is measured through the identification of the responder patients, defined as those showing an improvement of at least 20% in the ALSFRS-R slope during the 18-month randomization period compared to the 3-month lead-in period. Slope coefficients for ALSFRS-R decline will be calculated separately for the lead-in period and the treatment period using the linear regression model.

The secondary efficacy outcomes are presented in Table 2. The safety and tolerability of TUDCA will be evaluated through the assessment of adverse events, concomitant treatments, physical examinations, vital signs, and routine laboratory tests (hematology and biochemistry).

**Table 2:** Secondary and exploratory outcomes

Variable	Description
**Clinical**	
ALSFRS-R score (*ALSFRS-R)*	ALS Functional Rating Scale Revised consists of 12 questions, each scored out of 4 points. A total score is calculated by summing the scores from each question with a maximum score of 48 (better performance, i.e., absence of the measured symptoms of ALS) and a minimum score of 0 (worst performance)
Survival time measured by death or respiratory insufficiency	Survival status is recorded at the end of study as well as respiratory insufficiency defined as tracheostomy or the use of non-invasive ventilation for ≥ 22 h per day for ≥ 10 consecutive days. Survival time is defined as the time elapsed between randomization and death from any cause and tracheostomy, whichever occurs first
Forced vital capacity score (FVC)	The index of respiratory function expressed as a percentage of the expected value is used to indicate potential respiratory compromise
Muscle force assessed by the Medical Research Council (MRC) scale	MRC score is a summation of the strength of 6 muscle groups tested on both sides ranging from 0 (paralysis) to 60 (normal strength)
**Health-related quality of life**	
Amyotrophic Lateral Sclerosis Assessment Questionnaire (*ALSAQ-40*)	ALSAQ-40 score is a patient self-report health status PRO based on 40 questions specifically used to measure the subjective well-being of patients with ALS in a scale from 0 (best possible health status) to 100 (worst possible)
EQ-5D scale score (*EQ-5D-5L*)	The 5-level EQ-5D version is a self- assessed health-related quality of life (HRQoL) questionnaires based on 5 questions ranging from 0 (worst health) to 100 (best health)
**Biomarker**	
Creatinine level	Plasma creatinine is a breakdown product of creatine phosphate and it is related to muscle mass, expressed in μmol/L or micromol/L
Neurofilament level	Value of neurofilaments (light chain and phosphorylated heavy chain) in the CSF and serum resulting as a mean from triplicate measurements expressed in pg/ml
MMP-9 expression	The expression of matrix metalloproteinase-9 in serum is a mean of at least two duplicates, expressed in pg/ml

### Analysis method for primary and secondary outcomes

In the primary analysis, the effect of treatment on the primary endpoint will be measured by means of the odds ratio estimated through an unadjusted logistic regression model including a dummy variable for treatment group. Moreover, as part of the sensitivity analyses, a multivariable logistic regression model will be performed. Survival outcome will be analyzed using Kaplan–Meier survival analysis and log-rank test. Univariate and multivariable Cox proportional hazards models will also be applied. Additionally, the joint modeling omnibus test [13] will be used to assess the simultaneous effect on ALSFRS-R and survival. The longitudinal secondary endpoints (ALSFRS-R, ALSAQ-40, FVC, EQ-5D scale, MRC scale, and biomarkers) measured on a continuous scale will be analyzed using a multivariable linear mixed effect model (LME) to estimate the mean difference in the rate of decline between TUDCA and placebo over 18 months. The following covariates will be included in the multivariable models: age, gender, type of ALS, and region of initial site of diagnosis.

Country will be included as a random factor in the LME models. Clustered standard errors by country will be used in logistic and Cox regression models to obtain robust estimates. All primary and secondary analyses will be carried out on both ITT and per-protocol populations.

### Subgroup analyses

Exploratory subgroup analyses will be performed for the primary outcome in the ITT population, according to the following subgroup variables measured at randomization (month 0): site of onset, type of ALS (familial, sporadic), age (categorized according to the median), duration of the disease since symptom onset (categorized according to the median), gender, country. Moreover, an exploratory analysis will be conducted stratifying patients according to neurofilaments level [14]. The subgroup effect will be estimated including an interaction term for each subgroup variable at time in the logistic regression. The forest plot will be used to summarize subgroup analyses. The subgroup analyses will also be carried out for the change from baseline on ALSFRS-R by adding the treatment-subgroup interaction term in the MLE model.

### Missing data

#### Imputation rules and key sensitivity analyses

In the primary analysis, for each patient, all available ALSFRS-R scores will be included in the linear regression model to estimate the monthly decline. The following rules for handling missing data will be applied: (i) for deceased patients, as death is clearly a poor outcome and there is no consensus on an alternative score, the lowest score (zero) will be assigned at the time of death, and (ii) for withdrawals or LTF patients without a post-randomization assessment of the ALSFRS-R score, the monthly decline of ALSFRS-R will be imputed using the “nearest neighbor” procedure [15]. For each of these subjects, we will identify the five other participants within the same treatment group whose baseline ALSFRS-R scores were closest to the baseline score of the patient. Then, the largest monthly ALSFRS-R decline observed among the identified neighbors (worst-case imputation) will be used for imputation. All missing data will be imputed within treatment groups defined by the randomized treatment assignment. As sensitivity analysis, within the nearest neighbor procedure, the “bestcase imputation” will also be carried out imputing the smallest observed monthly decline.

#### Multiple imputation-based sensitivity analyses

We intend to calculate the post-randomization slope only after imputing missing ALSFRS-R data in the 18 months of treatment period and then categorize participants into responders/non-responders and finally conduct logistic regression analysis to estimate the effect of TUDCA. This approach is supported by simulation studies conducted by Floden and Bell [16]. A first sensitivity analysis will be carried out using the multiple imputation (MI) technique under the missing at random (MAR) assumption to deal with missing ALSFRS-R data. The imputation model will include the treatment group, the prognostic baseline characteristics, and ALSFRS-R at baseline. For patients deceased or withdrawn without a post-randomization assessment, the same rules specified for primary analysis will be applied. Moreover, the imputation of missing data under the missing not at random (MNAR) assumption considering the reason for missingness will be carried out using the delta-based MI method. A range of penalties by reason of missingness (study termination due to adverse events AE and death of patients) will be considered. All other missing data will be imputed under MAR assumption, applying a null penalty. For withdrawals without a post-randomization assessment, the same rules specified for primary analysis will be used. A total of 50 imputations for each analysis will be performed, which should provide adequate precision since the amount of missing data is not expected to be large [17]. Results from different imputed datasets will be combined using Rubin’s rule.

### Supporting analysis

Based on suggestions received from the EMA Scientific Advice Working Party on 28 February 2017, we will also consider a definition of responders with a threshold of 25%. The same model specified for the primary analysis will be applied.

### Additional analyses to account for the COVID-19 pandemic

Prior to the COVID-19 pandemic, the primary analysis was planned using an ITT approach, by properly accounting for missing data and premature study termination. Despite the occurrence of the COVID-19 pandemic, the original primary objective of TUDCA-ALS study remains unchanged, implying that the estimate of TUDCA effect is not confounded by COVID-19 pandemic related disruptions. However, COVID-19-related intercurrent events need to be properly accounted for to ensure an appropriate interpretation of the trial results [18]. Following the recommendation received on December 1, 2022, after the mid-term review of the project by the European Commission, additional sensitivity and supplementary analyses have been planned in accordance with the ICH E9 (R1) Addendum on Estimands and Sensitivity Analysis in Clinical Trials [19].

In application of the COVID-19 amendment, changes have been made to adapt the electronic case report forms for the study visits performed after February 3, 2020. The main changes include the implementation of telemedicine visits, the specification of missing data due to COVID-19, collecting information on COVID-19 protocol deviations, COVID-19 vaccination, and study termination due to COVID-19. Information on COVID-19-related infections, therapy, and death are expected to be reported in the descriptions of protocol deviations, as well as in the form on concomitant medication and adverse events. All these data will be used to perform additional analyses in accordance with the specific EMA guidelines on the implications of coronavirus disease (COVID-19) on methodological aspects of ongoing clinical trials [20].

#### Assessing the impact of COVID-19 pandemic on trial conduction

To describe the impact of COVID-19 pandemic on trial conduction, with the aim of excluding treatment-specific data patterns by reason due to COVID-19, the following analysis will be performed by treatment group. Baseline characteristics will be summarized by the enrolment period categorized in pre-pandemic, pandemic with restrictive measures, and post-pandemic. The number of visits conducted via telemedicine (including audio-visual connections and telephone contacts), missing data, protocol deviations, end of study by reason, COVID-19 infections, concomitant medications for COVID-19, and death due to COVID-19, will be reported overall and by treatment group. A time to occurrence analysis will be conducted for COVID-19-related protocol deviations using Kaplan-Maier curves and the log-rank test.

#### Assessing the impact of the COVID-19 pandemic on treatment effect

Even if self-administered ALSFRS-R collected through telemedicine is a validated approach [21–23], an assessment of the association between on-site and telemedicine visits will be evaluated by comparing the distributions of ALSFRS-R measurements by the modality of visit conduction (in-person or telemedicine) stratifying for treatment group and study visit. The modality of visit conduction will be included in the multivariable LME model for ALSFRS-R as a fixed effect. To assess the impact of the pandemic on the ALSFRS-R slope, the pandemic time-period and the infection status will also be included in the multivariable LME model as fixed effects. The interaction term between treatment and pandemic time-periods will be included, and the treatment effect by pandemic periods will be reported. Additionally, an estimate of treatment effect will be obtained through inverse probability treatment weighting to assess the potential confounding effect of COVID-19 intercurrent events [24].

#### Sensitivity analyses for handling missing data in the primary endpoint

Missing data are expected to increase during the trial due to the COVID-19 pandemic. In the primary and key sensitivity analyses, pandemic-related missing data will be treated as all the other missing data.

In these additional analyses, according to the estimand framework [19], the treatment policy strategy is adopted for COVID-19 intercurrent events. In such approach, we consider the pandemic irrelevant in the effect estimate, and all collected data are used in the analyses. All missing data will be imputed by MI under MAR assumption, by including also COVID-19 infection status and pandemic time-period in the imputation model [25]. Moreover, under the assumption that the pandemic (restrictive measures, lockdown and infections) could negatively affect the outcome, the analysis under MNAR assumption will be repeated, conducting a tipping point analysis for COVID-19-related missing data. MI analysis will be conducted using the number of imputations and the pooling rule previously specified.

#### Sensitivity analysis for the secondary survival endpoint

As the pandemic may affect the survival probability, the proportional hazards assumption will be checked by visual assessment of Kaplan–Meier curves, log(− log) plots, and testing of scaled Schoenfeld residuals. If the assumption of proportional hazards between treatment groups is not verified, the ratio of restricted mean survival time between groups will be provided [18, 26, 27]. The infection status and the pandemic time-period will be included in the multivariable Cox model. Moreover, in case the number of censoring due to COVID-19 is not negligible, the treatment effect will be evaluated by using the inverse probability of censoring weighting method. The weights will be calculated by a logistic regression model considering baseline characteristics and time-dependent covariate such as the infection status and pandemic time-period [28, 29].

Finally, due to the reduction in sample size and consequently the decrease in power, an analysis assessing the association of the survival time with the disease progression (ALSFRS-R slope) will be conducted. We will perform the Cox multivariable regression, in which the primary exposure is the slope of ALSFRS-R score. The analysis will also be repeated considering the responder status as the main exposure.

### Safety/harms

All AEs, distinct by severity and relationship with the randomized drug, will be documented and listed. The number and percentage of AEs will be reported, per treatment arm and overall, along with the number and percentage of participants who experienced at least one AE. Moreover, the number of concomitant medications and changes from baseline in vital signs, routine biochemistry and hematology analyses, and in respiratory endpoints will be analyzed. Discrete safety endpoints will be compared using a chi-squared test. The denominator will be the safety population. Continuous safety endpoints will be compared using a *t*-test for parametric variables or the Mann–Whitney test for non-parametric variables. The assumption of normal distribution will be checked using Shapiro–Wilk’s test.

Furthermore, AEs will be described according to the different pandemic periods [18].

#### Statistical software

Analyses will be carried out by the STATA 17 and R software (version 4.3.0).

## Discussion

The TUDCA-ALS incorporates the design and the experience of the previous phase II TUDCA study [5], with strengthened endpoints and the addition of innovative biomarker analysis. The responder analysis provides an innovative clinical design in ALS studies that overcomes several methodological difficulties observed in the classical parallel-group design [4]. In the earlier phase II study, responder status was defined as a minimum 15% improvement in the ALSFRS-R progression slope in the double-blind treatment phase compared to the lead-in phase. For this trial, the threshold was increased to 20%, based on suggestions received by the EMA Committee for Orphan Medicinal Products. Additionally, a supportive analysis is planned using a threshold of 25%, which was deemed clinically meaningful according to a survey conducted among experienced ALS clinical investigators [30].

The TUDCA-ALS trial, like many clinical trials worldwide, has been significantly impacted by the COVID-19 pandemic [31]. Clinical sites started recruiting patients in February 2019 across the seven involved countries, and the first patient was randomized in May 2019. The first cases of COVID-19 in Europe were registered in January 2020, with an initial outbreak in Italy and subsequent outbreaks in other European countries. The WHO declared COVID-19 a global pandemic on March 11, 2020. This unforeseen event required the prompt implementation of specific and unprecedented mitigation measures. Several interventions were implemented aimed at ensuring the completion of the trial, such as sample size reduction, introduction of telemedicine for in-person visit replacements, and the collection of information on COVID-19-related intercurrent events [9].

In the planning of sensitivity analyses, the issue of the potential impact of the pandemic on trial conduction and interpretation of results was taken into account. Special consideration is needed as some studies reported the effect of COVID-19 and of the restrictive measures on the progression of ALS [32, 33]. Moreover, persons with ALS can be at a higher risk of COVID-19 infection and its complications [34, 35]. In addition, country-specific measures were implemented to address and mitigate the COVID-19 pandemic, such as government-enforced closures, temporary cessation of study-related activities at sites, and vaccination campaigns, with differences by countries that could impact the study. Following EMA guidelines [20] and recommendations from the literature [18, 25], additional analyses were introduced to evaluate the effect of these changes on the conduct of the study and its results. Sensitivity analyses were also planned to handle missing data due to COVID-19, under different assumptions. Sensitivity analyses for the survival outcome were included, considering that the reduced sample size cannot guarantee the same power initially specified. All the proposed analyses aim to provide a full understanding and interpretation of the trial results.

## Conclusion

This paper presents the details of the statistical analysis to be conducted in accordance with the relevant guidelines and regulations. By incorporating sensitivity and supplementary analyses in the SAP, it will be possible to record the influence of COVID-19, both in terms of intercurrent events and treatment effect. The results of the pre-specified analyses will be reported in the clinical study report in order to minimize the outcome reporting bias.

### Trial status

Recruitment status: closed on 23 December 2021 (last randomization on 04 April 2022).

Recruitment start date: 22 February 2019 (first randomization on 20 May 2019).

### Supplementary Information

**Additional file 1.** Statistical Analysis Plan (SAP) Cheklist.

## Data Availability

Not applicable.

## Abbreviations

AE: Adverse event
ALS: Amyotrophic lateral sclerosis
ALSAQ-40: ALS Assessment Questionnaire-40
ALSFRS-R: ALS functional rating scale – revised version
CONSORT: CONsolidated Standards of Reporting Trials
COVID-19: Coronavirus disease of 2019
CSF: Cerebrospinal fluid
EMA: European Medicine Agency
EQ-5D-5L: Euro Quality of Life 5 dimensions scale 5-level version
FVC: Forced vital capacity
LME: Linear mixed-effect
LTF: Lost to follow-up
ICH: International Council for Harmonisation
ITT: Intention-to-treat
MAR: Missing at random
MI: Multiple imputation
MNAR: Missing not at random
MMP-9: Matrix metalloproteinase 9
MRC: Medical Research Council
SAP: Statistical analysis plan
TUDCA: Tauroursodeoxycholic acid
